# Evaluating the composition and processing potential of novel sources of Brazilian biomass for sustainable biorenewables production

**DOI:** 10.1186/1754-6834-7-10

**Published:** 2014-01-18

**Authors:** Marisa A Lima, Leonardo D Gomez, Clare G Steele-King, Rachael Simister, Oigres D Bernardinelli, Marcelo A Carvalho, Camila A Rezende, Carlos A Labate, Eduardo R deAzevedo, Simon J McQueen-Mason, Igor Polikarpov

**Affiliations:** 1 Instituto de Física de São Carlos, Universidade de São Paulo, Caixa Postal 369, São Carlos SP 13560-970, Brazil; 2 CNAP, Department of Biology, University of York, York, Heslington YO10 5DD, UK; 3 Embrapa Cerrados, Genética e Melhoramento de Forrageiras, Br 020, Km 18 – Cx. P. 08223, Planaltina DF 73301-970, Brazil; 4 Instituto de Química, Universidade de Campinas, Caixa Postal 6154, Campinas SP 13083-970, Brazil; 5 Laboratório Max Feffer de Genética de Plantas, Departamento de Genética -ESALQ, Universidade de São Paulo, Caixa Postal 83, Piracicaba SP 13418-900, Brazil; 6 Centro Nacional de Pesquisa em Energia e Materiais, Laboratório Nacional de Ciência e Tecnologia do Bioetanol (CTBE), Campinas, SP, Brazil

**Keywords:** Bioethanol, *Brachiaria brizantha*, Brazilian grasses, Chemical composition, Enzymatic saccharification, Eucalyptus barks, *Panicum maximum*, *Pennisetum purpureum*, Pretreatments, Scanning electron microscopy, Sugarcane bagasse

## Abstract

**Background:**

The search for promising and renewable sources of carbohydrates for the production of biofuels and other biorenewables has been stimulated by an increase in global energy demand in the face of growing concern over greenhouse gas emissions and fuel security. In particular, interest has focused on non-food lignocellulosic biomass as a potential source of abundant and sustainable feedstock for biorefineries. Here we investigate the potential of three Brazilian grasses (*Panicum maximum*, *Pennisetum purpureum* and *Brachiaria brizantha*), as well as bark residues from the harvesting of two commercial Eucalyptus clones (*E. grandis* and *E. grandis x urophylla*) for biofuel production, and compare these to sugarcane bagasse. The effects of hot water, acid, alkaline and sulfite pretreatments (at increasing temperatures) on the chemical composition, morphology and saccharification yields of these different biomass types were evaluated.

**Results:**

The average yield (per hectare), availability and general composition of all five biomasses were compared. Compositional analyses indicate a high level of hemicellulose and lignin removal in all grass varieties (including sugarcane bagasse) after acid and alkaline pretreatment with increasing temperatures, whilst the biomasses pretreated with hot water or sulfite showed little variation from the control. For all biomasses, higher cellulose enrichment resulted from treatment with sodium hydroxide at 130°C. At 180°C, a decrease in cellulose content was observed, which is associated with high amorphous cellulose removal and 5-hydroxymethyl-furaldehyde production. Morphological analysis showed the effects of different pretreatments on the biomass surface, revealing a high production of microfibrillated cellulose on grass surfaces, after treatment with 1% sodium hydroxide at 130°C for 30 minutes. This may explain the higher hydrolysis yields resulting from these pretreatments, since these cellulosic nanoparticles can be easily accessed and cleaved by cellulases.

**Conclusion:**

Our results show the potential of three Brazilian grasses with high productivity yields as valuable sources of carbohydrates for ethanol production and other biomaterials. Sodium hydroxide at 130°C was found to be the most effective pretreatment for enhanced saccharification yields. It was also efficient in the production of microfibrillated cellulose on grass surfaces, thereby revealing their potential as a source of natural fillers used for bionanocomposites production.

## Background

The production of biorenewables, particularly liquid biofuels, from lignocellulosic biomass has become a strategic research area because it holds the potential to improve energy security, decrease urban air pollution and reduce CO_2_ accumulation in the atmosphere [1,2]. In turn, the biorefining platforms required for biofuels production present an opportunity to stimulate new markets for the agriculture sector and increase domestic employment, contributing to the development of emerging economies [3].

In Brazil, the production of first-generation ethanol from sugarcane juice (sucrose) has made the country a leading producer of biofuels. At present, approximately 90% of the automobiles made in Brazil are dual-fuel [4]. In 2006 to 2007, Brazilian ethanol production reached 18 billion liters, supplying the domestic demand and producing an excess of 3.5 billion liters for export. International targets for a reduction in CO_2_ emission combined with high oil prices are driving an increase in global bioethanol production, which is predicted to reach 43 billion liters in 2025. Meeting this demand will require a 130% increase in the area of cultivated sugarcane [5]. In this context, second-generation biofuels, which use, for example, biomass feedstocks, agricultural wastes and wood residue, represent an efficient and complementary approach to increase liquid biofuel production. The adoption of second-generation bioethanol production from lignocellulosic biomass is attractive from a number of perspectives. By making use of all available biomass, such approaches can improve the carbon footprint of biofuels further, as well as increasing the yield of ethanol per hectare and providing a means to sustain the operation bioethanol plants throughout the year, instead of their current seasonal operation [2,6,7].

The diversity of climates and agricultural conditions in Brazil enables the growth of a large diversity of lignocellulosic materials. The management of this primary productivity can be driven towards high output/low input systems, which are optimal for second-generation fuels. In addition, Brazilian agriculture provides large volumes of lignocellulosic residues that could be used for biofuel production.

Among these residues, sugarcane bagasse is the most promising Brazilian feedstock for lignocellulosic ethanol production, being a by-product of first-generation ethanol production and therefore available in large amounts at sugarcane mills. According to the Brazilian Ministry of Agriculture, sugarcane production for 2012 to 2013 is estimated to reach 650 million tons and each ton of cane milled generates approximately 260 kg of bagasse, which is used mainly to co-generate the electricity needed for the operation of the mill [8,9]. Thus, to date, most research has focused on sugarcane bagasse as a feedstock for second-generation biofuel production, with the potential to increase bioethanol production in Brazil by one third.

However, increasing Brazilian bioethanol production by one third will be insufficient to meet future demand, and it is clear that consideration of other sources of biomass is necessary. Brazil has around 6.5 million hectares of cultivated forest, among which 4.8 million hectares are occupied by eucalyptus and the remaining fraction by pine. The forest industry is a source of large quantities of lignocellulosic residues such as bark and branches, which can potentially be used for second-generation bioethanol, but are currently left in the field [10,11]. Approximately 30% of the total biomass produced in Brazil by eucalyptus forestry is lost as residues, when the trees are harvested at the end of a seven-year cycle. The bark proportion in eucalyptus forestry can reach between 10% and 12% of the total biomass harvested, which represents a volume of 15 to 25 ton/ha/year [12-14], making this a promising feedstock for bioethanol production [15].

The diversification of feedstock for lignocelluloses-derived fuels requires an innovative approach that expands beyond the agricultural wastes. Perennial grasses, such as miscanthus and switchgrass, have been proposed as key bioenergy crops in Europe and the US, based on their low input and marginal land requirements. These biomass grasses could also make a substantial contribution within the Brazilian energy matrix, serving as an alternative to sugarcane inter-season, when there is no bagasse production. Although switchgrass and miscanthus could be used in Brazil, there are also a number of other candidate biomass grasses that are already established and characterized from an agronomical point of view. Brazil has the fourth largest worldwide cultivated pasture area, reaching around 174 million hectares; around 30% of national territory, distributed throughout the country [16,17]. The tropical climate in Brazil supports the efficient growth of a range of grasses with high productivity, for example, from the genus *Brachiaria*, *Panicum*, *Pennisetum* and *Cynodon*, which are very important for Brazilian beef and dairy cattle production. *Brachiaria* was first introduced to Brazil 15 years ago, and today occupies around 70% of total pasture area, followed by *Panicum*, which occupies approximately 10%. Initial studies have shown promising averages of productivity yields (dry mass) for different perennial grasses species compared to sugarcane, for example: *Pennisetum purpureum* (35 ton/ha), *Panicum maximum* (30 ton/ha) and *Brachiaria brizantha* (20 ton/ha), compared to sugarcane at 30 ton/ha [18].

In this paper, we have investigated and compared the potential of three grasses (*Panicum maximum*, *Pennisetum purpureum* and *B. brizantha*) and eucalyptus barks (from *Eucalyptus grandis* and the hybrid *E. grandis x urophylla*) against sugarcane bagasse as feedstocks for bioethanol production. We examined the general composition of these potential feedstocks and compared their suitability for processing to produce sugars for fermentation under a range of conditions. The aim of this characterization was to increase the range of potential feedstocks for Brazilian biofuel production to include sustainable biomass sources outside the human food chain.

## Results and discussion

The development of second-generation biofuels requires a diverse set of feedstocks that can be grown sustainably and processed cost effectively. In particular, many biofuel production plants operate seasonally and stand idle for several months of the year, and this is unsatisfactory as it denotes an inefficient use of capital as well as providing only intermittent employment for workers. One way to avoid discontinuous biofuel production is to use a wider range of biomass sources that may be available during the current idle periods. Here, the potential of three widely grown, high-yielding Brazilian grasses, as well as the bark from two commercial eucalyptus clones, was investigated and compared with sugarcane bagasse, the most widely used biomass for bioethanol production. The biomasses were subjected to a range of pretreatment conditions to evaluate their effects on cellulose accessibility and enzymatic digestibility, as well as the levels of inhibitors produced.

### Biomass composition

For all six feedstocks, the biomass composition was analyzed for soluble extractives, silicon, cellulose, hemicellulose and lignin contents (Table 1). Bagasse is extensively washed during the commercial extraction of sucrose for first-generation ethanol and, as expected, the sequential extraction using organic solvents revealed a lower soluble content in sugarcane bagasse (3.39 ± 1.26%). *Panicum maximum* and *Pennisetum purpureum* showed 5.23 ± 2.37% and 5.70 ± 2.25% of solubles, respectively, whereas *B. brizantha* had more than twice as much soluble material (12.41 ± 3.69%) as all three of these feedstocks. The amount of solubles extracted from eucalyptus bark (approximately 27%) was much higher, which correlates with previous results published by our research group [15].

**Table 1:** Biomass composition of raw Brazilian biomasses

**Biomass**	**Solubles**	**Silicon**	**Cellulose**	**Hemicellulose**	**Lignin**	**Maximum theoretical ethanol yield (L/dry ton)**^**a**^	**Productivity** **(ton/ha)**^**b**^	**Maximum theoretical ethanol yield (L/ha)**^**c**^
	**(%)**	**(%)**	**(%)**	**(%)**	**(%)**			
Sugarcane bagasse	3.39 ± 1.26	0.44 ± 0.03	39.44 ± 1.21	27.45 ± 2.08	27.79 ± 1.39	282.62	30	8,478.6
*Panicum maximum*	5.23 ± 2.37	1.07 ± 0.01	39.87 ± 1.97	26.62 ± 1.46	25.36 ± 1.06	285.70	30	8,571.0
*Pennisetum purpureum*	5.70 ± 2.25	0.85 ± 0.01	45.97 ± 3.10	27.03 ± 1.02	22.80 ± 1.26	329.41	35	11,529.4
*Brachiaria brizantha*	12.41 ± 3.69	1.38 ± 0.06	43.48 ± 1.84	23.23 ± 3.16	23.09 ± 0.73	311.57	20	6,231.4
*E. grandis* bark	28.29 ± 3.43	0.03 ± 0.01	39.54 ± 1.10	18.84 ± 4.11	21.57 ± 1.59	283.34	25	7,083.5
*E. grandis* x *urophylla* bark	28.13 ± 2.20	0.03 ± 0.01	40.36 ± 4.31	16.45 ± 3.05	22.18 ± 2.22	289.21	25	7,230.3

^a^ Calculated considering the total cellulose conversion in the sample, according to the National Renewable Energy Laboratory standards [19]. ^b^ Source: [11,18]. ^c^ Calculated with base on total cellulose conversion in the sample and average Brazilian biomasses productivity (ton/ha).

Silicon is considered an important macronutrient for plant growth and development, particularly in grasses, where it is important for tissue strength and resistance to environmental stress and pathogens [20]. Generally, silicon represents the major mineral content in grasses and can accumulate up to 15% in some species such as rice, where it mostly occurs as amorphous silica with some silicon dioxide [21]. Silicon can cause problems in certain industrial processes [22,23], so it is pertinent to assess silicon levels in potential biomass sources. Quantification of silicon by X-ray fluorescence (XRF) shows that the perennial grasses, *B. brizantha* (1.38 ± 0.06%), *Panicum maximum* (1.07 ± 0.01%) and *Pennisetum purpureum* (0.85 ± 0.01%) contain higher silicon levels than sugarcane bagasse (0.44 ± 0.03%) (Table 1), whereas silicon levels in bark were much lower (0.03 ± 0.01 for both clones). The inorganic fraction of eucalyptus barks is composed mainly of calcium crystals in the form of calcium oxalate or carbonate [24,25]. The higher amount of silicon in the perennial grasses was accompanied by the presence of phytoliths, classified as panacoids, on the biomass surface, as observed by scanning electron microscopy (Additional file 1). Phytoliths are microscopic silica bodies that precipitate in or between cells of living plant tissues and are especially abundant, diverse and distinctive in the grass family [26].

Levels of cellulose, hemicellulose and lignin were determined biochemically and the results are shown in Table 1. Lignin is a complex polymer of phenyl propane units (*p*-coumaryl, coniferyl and sinapyl alcohol) that acts as a cementing and waterproofing agent. It is generally considered to be a barrier to the efficient saccharification of biomass [27].

Lignin content varied from 27.79% in sugarcane bagasse to approximately 22% in eucalyptus bark, with intermediate values in the perennial grasses. The hemicellulose fraction of the feedstocks was higher in the grasses, varying from 27% in sugarcane bagasse to 23% in *B. brizantha,* and was considerably lower in eucalyptus bark at about 19% and 16% for *E. grandis* and *E. grandis x urophylla* bark, respectively. Cellulose content, on the other hand, was highest in *Pennisetum purpureum* (46%), followed by *B. brizantha* (43%), whereas sugarcane bagasse, *Panicum maximum* and both eucalyptus barks showed a cellulose content of approximately 40%.

The carbohydrate fraction of these biomasses represents their potential for the biochemical conversion of sugars into lignocellulosic ethanol. Using the standard equations from the National Renewable Energy Laboratory [19] and considering total conversion of the cellulosic fraction, the potential ethanol yield (L/dry ton) for each biomass was calculated and is presented on Table 1. The highest ethanol yield (329.41 L/dry ton) was found for *P. purpureum*, reflecting its high cellulose content. Considering the biomass productivity values taken from published literature (also shown on Table 1) [11,18], it was possible to estimate the total theoretical ethanol yield (L/ha) for each of the evaluated feedstocks. It must be emphasized that these are simple approximate indications because the data are derived from a range of different crop yield studies and based on theoretical fermentation yield calculations. However, these values suggest that *Pennisetum purpureum* looks particularly promising due to its higher biomass productivity and cellulose content (around 35 ton/ha), which suggests a theoretical ethanol yield of more than 11,500 L/ha. This compares favorably with the first generation Brazilian bioethanol productivity from sugarcane juice, at around 6,000 L/ha [28]. As has been previously discussed, the yield of ethanol from bark could be higher than reported here, as considerable amounts of sugar occur in the soluble extractives (not included in this calculation), but this depends on how soon after harvest the bark is processed [29].

### Immunolabeling of hemicellulose polysaccharides

**Figure 1:**
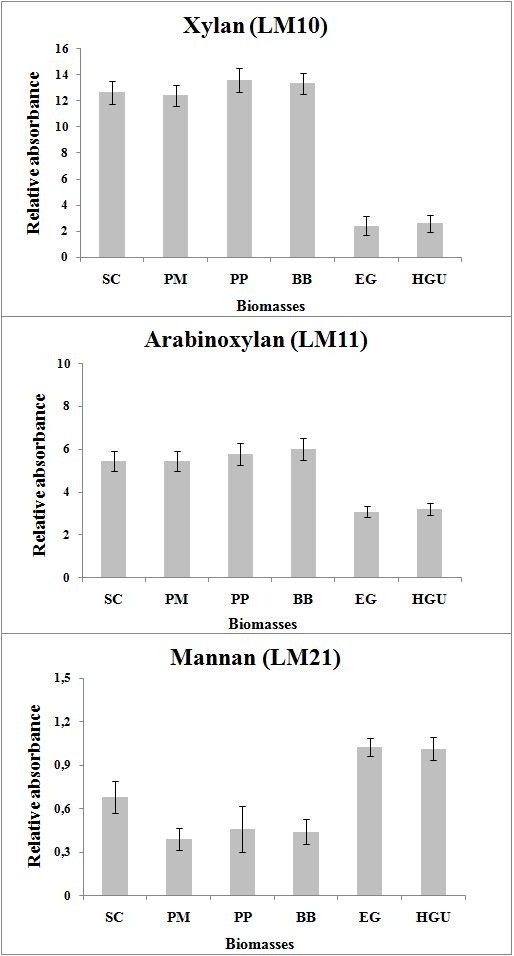
**ELISA of xylans (LM10 and LM11) and mannan (LM21) polysaccharides on hemicellulose fraction from Brazilian grasses and *Eucalyptus *barks.** SC, sugarcane bagasse; BB, *Brachiaria brizantha*; EG, *Eucalyptus grandis* bark; HGU, bark of hybrid between *Eucalyptus grandis* x *urophylla*; PM, *Panicum maximum*; PP, *Pennisetum purpureum*. Values expressed as relative absorbance to the positive control (xylan - μg/mL).

The composition of the hemicellulosic fraction of a biomass feedstock is one of the key determinants in selecting a choice of process for conversion. Paradoxically, the C5 sugars present in hemicelluloses represent both a hurdle for fermentation and a source of platform chemical for added value products. A rapid and reliable way to evaluate the relative content of key polysaccharides in the hemicellulosic fraction is by using immunobased techniques. Here, we used an ELISA-based approach to compare the six biomasses for their xylan, arabinoxylan, mannan, galactomannan, and glucomannan content. The hemicellulosic fraction was extracted with sodium hydroxide and analyzed by ELISA using the following antibodies: LM10 (recognizes unsubstituted and relatively low-substituted xylans, and has no cross-reactivity with wheat arabinoxylan), LM11 (recognizes unsubstituted and relatively low-substituted xylans, but can also accommodate more extensive substitution of a xylan backbone and binds strongly to wheat arabinoxylan) and LM21 (binds effectively to β-(1 → 4)-manno-oligosaccharides from DP2 to DP5, displays a wide recognition of mannan, glucomannan and galactomannan, and has no known cross-reactivity with other polymers) [30-32]. Figure 1 shows that the hemicellulose fraction from the grasses gave strong signals with LM10 and 11 antibodies indicating a high content of xylans and arabinoxylans as typically seen in grasses, with lower signals for the mannan-detecting LM21 antibody. The hemicellulose fraction of sugarcane bagasse, *Panicum maximum*, *Pennisetum purpureum* and *B. brizantha*, after an initial 40-times dilution, showed a relative absorbance more than 12 times higher than the absorbance found for the positive control (10 μg/mL xylan). By contrast, xylan levels appeared lower in the hemicelluloses fraction from eucalyptus barks at the same initial dilution. The relative absorbance for the barks was around 2.5 times the positive control when LM10 was used.

The hemicellulose fractions from all biomasses were also diluted 40 times before the immunolabeling assays using LM11. However, the relative absorbance found for the grasses were reduced by approximately half (around 5.7 times the positive control), indicating lower arabinoxylan content when compared to xylans. By contrast, the relative absorbance for the eucalyptus barks increased by around 3.2 times, suggesting a higher content of derived arabinoxylans on its hemicellulose fraction.

When LM21 was used, the initial hemicelluloses fractions dilution needed was only 20 times, indicating a lower content of mannans polysaccharides for all six feedstocks when compared to xylans and arabinoxylans. The relative absorbance found for the three grasses were lower (around 0.4 times) than the positive control (galactomannan, 10 μg/mL), while for both Eucalyptus barks it was approximately the same as the control. The relative absorbance for sugarcane bagasse was 0.7 times that of the positive control. The higher relative absorbance found for eucalyptus barks suggests a higher content of mannans compared to the grasses and sugarcane bagasse.

### Effect of pretreatments on the composition of different feedstocks

There is consensus regarding the need for a pretreatment to remove and/or modify the matrix of lignin and hemicellulose surrounding the cellulose fraction, to enable efficient enzymatic saccharification of cellulose [33]. However, the complexity and heterogeneity found in the lignocellulosic biomass of different species makes it is advisable to optimize a pretreatment for each feedstock, to enable maximum saccharification whilst avoiding the generation of inhibitors of fermentation, such as furfurals. Ideally, a pretreatment should preserve the hemicellulose fraction, limit inhibitor formation, minimize the energy input, be cost-effective, warrant the recovery of high value-added co-products (for example, lignin) and minimize the production of toxic waste [12,34].

**Figure 2:**
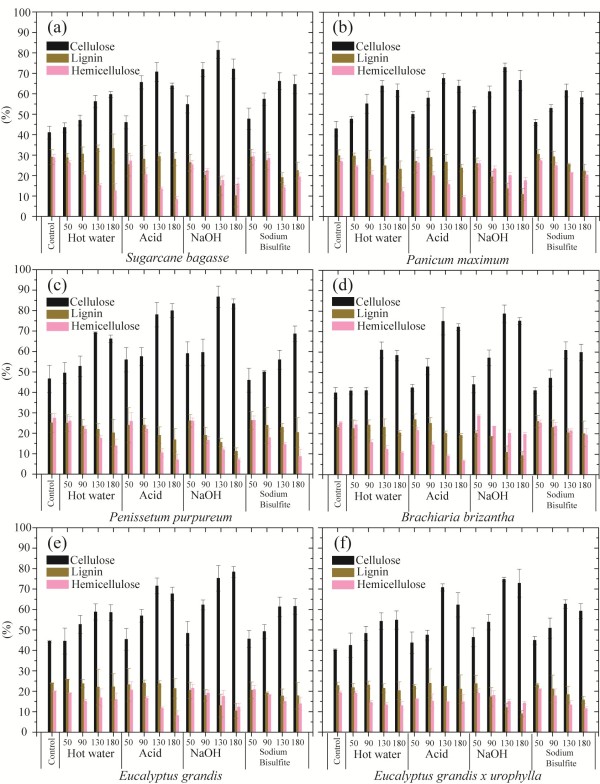
**Chemical composition of non-pretreated and pretreated biomasses. (a)** Sugarcane bagasse; **(b)***Panicum maximum*; **(c)***Pennisetum purpureum*; **(d)***Brachiaria brizantha*; **(e)***Eucalyptus grandis* bark; **(f)** bark of *E. grandis x urophylla*. Pretreatment types and temperatures are indicated.

Since the composition of different biomasses affects the efficiency of processing, it will also influence the choice of pretreatments required to maximize the recovery of sugars. To evaluate this particular issue, we pretreated the six feedstocks under acid, alkaline, sulfite and hot water conditions over a range of temperatures. Figure 2 shows the averages of the three main components (cellulose, hemicellulose and lignin) content determined using different methods at microscale, as described in the Materials and Methods section. The standard deviations found for each of three components of the biomasses are also given in Figure 2.

Hot water pretreatment showed a similar effect over the chemical composition of the different biomasses, removing mainly the hemicellulose fraction. The lignin content remained fairly constant (varying between 27% and 23%), while the average cellulose content increased from around 40% to 60% as the temperature increased to 130°C (Figure 2). This enrichment in cellulose is a direct consequence of the removal of hemicellulose. However, at 180°C, the cellulose content was lower, possibly due to the production of degrading compounds such as furaldehydes, rather than a reduction in hemicelluloses removal at this temperature. On average, pretreatment at 180°C resulted in a reduction in the hemicelluloses fraction from approximately 25% (untreated feedstocks) to 13% (pretreatment at 180°C), ranging between 10.74 ± 0.62% in *B. brizantha* and 15.09 ±1.08% in *E. grandis* bark (Figure 2).

The acid pretreatment was highly efficient for hemicellulose removal, and an increase in temperature (up to 130°C) had a further positive effect when compared to hot water treatment. However, at 180°C, the degrading hemicellulose product, 2-furfuraldehyde, was detected for all three grasses, with a higher content in *B. brizantha* liquor fraction, and for *E. grandis* bark. At the highest temperature (180°C), higher cellulose losses were also observed, and the average cellulose content decreased to around 60% after acid pretreatment at 180°C, compared to 70% at 130°C. However, even with the increase in temperature, acid pretreatment was not sufficient for lignin removal (Figure 2). At the highest temperature applied in this study (180°C), approximately 20% of remaining dry matter was lignin.

The highest cellulose enrichment was observed in samples subjected to the alkaline pretreatment using sodium hydroxide, which removed higher quantities of both lignin and hemicellulose fractions. The average lignin content across all feedstocks was reduced from around 27% to 9% at 180°C. However, at this temperature, some cellulose losses were observed, particularly in sugarcane bagasse and *Panicum maximum*.

The chemical composition of biomasses submitted to treatment with sodium bisulfite at increasing temperatures was observed to be similar to hot water pretreatment. In all feedstocks, an increase in cellulose enrichment was observed until 130°C, reaching around 60%, with a maximum enrichment observed for sugarcane bagasse (66.1 ± 4.14%). At 180°C, a slight decrease in cellulose content was observed for sugarcane bagasse, *Panicum maximum* and the bark of *E. grandis x urophylla*. The cellulose fraction from *B. brizantha* and *E. grandis* remaining constant between 130°C and 180°C. Conversely, *Pennisetum purpureum* showed a gradual increase on its cellulose fraction until 180°C, reaching around 56% at 130°C and 68% at the highest temperature. The discrete cellulose enrichment observed after sulfite pretreatment is associated with a low removal of both hemicellulose and lignin.

**Figure 3:**
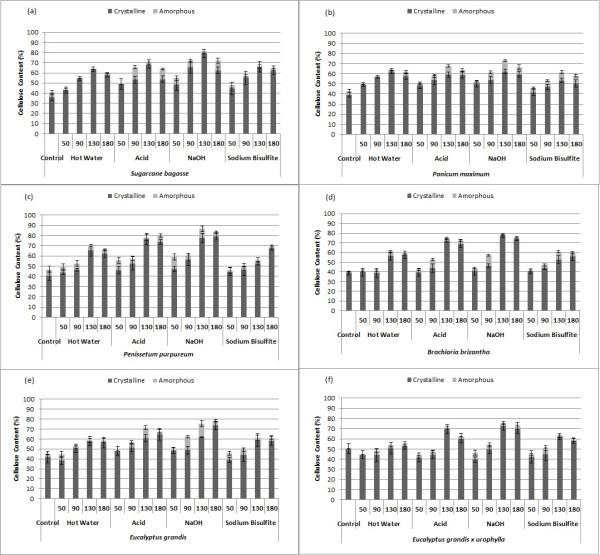
**Crystalline and amorphous cellulose content of pretreated samples and biomasses without soluble (control). (a)** Sugarcane bagasse; **(b)***Panicum maximum*; **(c)***Pennisetum purpureum*; **(d)***Brachiaria brizantha*; **(e)***Eucalyptus grandis* bark; **(f)** bark of *E. grandis x urophylla*. Pretreatment types and temperatures are indicated.

The content of amorphous and crystalline cellulose after different pretreatment conditions was determined by a chemical method and each fraction is shown in Figure 3. We observed a clear increase in the crystalline portion of the cellulosic fraction until 130°C for all species and all pretreatments used. At 180°C, however, some losses in the crystalline fraction could be observed, mainly after hot water and acid pretreatment for the grasses. Analysis of the amorphous content of control samples indicated a variation of between 2% and 13% of total cellulose content in this fraction. The highest amorphous content was observed for *Pennisetum purpureum* (approximately 6% of cell-wall composition), followed by sugarcane bagasse (about 5%). The lowest amorphous cellulose content was observed in *B. brizantha*. No clear correlation between pretreatment conditions and the amorphous cellulose fraction was determined. However, considering the glucose content in the soluble fraction from pretreatment, it is possible that at lower temperatures this fraction was mainly removed, while at higher temperatures there was also a degree of biomass amorphization.

**Figure 4:**
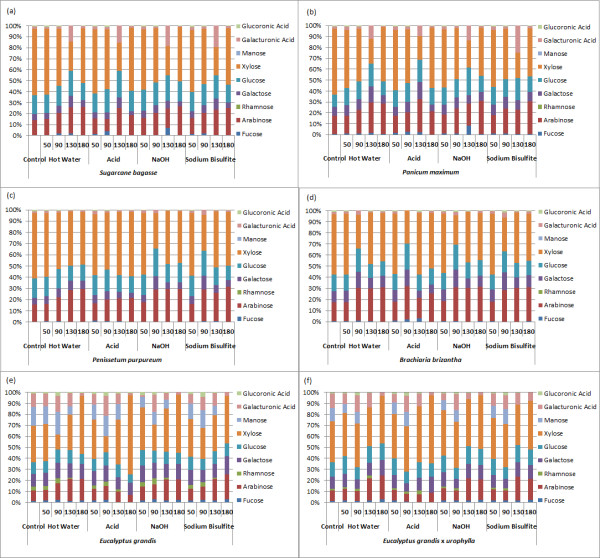
**Monosaccharide composition on the hemicellulose fraction of pretreated samples and biomasses without soluble (control). (a)** Sugarcane bagasse; **(b)***Panicum maximum*; **(c)***Pennisetum purpureum*; **(d)***Brachiaria brizantha*; **(e)***Eucalyptus grandis* bark; **(f)***E. grandis x urophylla* bark. Pretreatment types and temperatures are indicated.

Hemicellulose fractions were analyzed after pretreatment to evaluate the changes in monosaccharide composition (Figure 4). Sugarcane bagasse, *Panicum maximum*, *Pennisetum purpureum* and *B. brizantha* showed a similar composition in the hemicellulose fraction, composed mainly of xylose, arabinose and glucose, followed by lower amounts of galactose and fucose. The hemicellulose fraction from eucalyptus barks was more heterogeneous, with lower xylose content when compared to the grasses (Figure 4). Barks showed a high amount of mannose and rhamnose, not detected in the grasses. These results were in agreement with the ELISA results, which indicated a higher content of xylans and arabinoxylans in the grasses and a significant level of mannans in the eucalyptus bark feedstocks.

### Solid-state nuclear magnetic resonance

**Figure 5:**
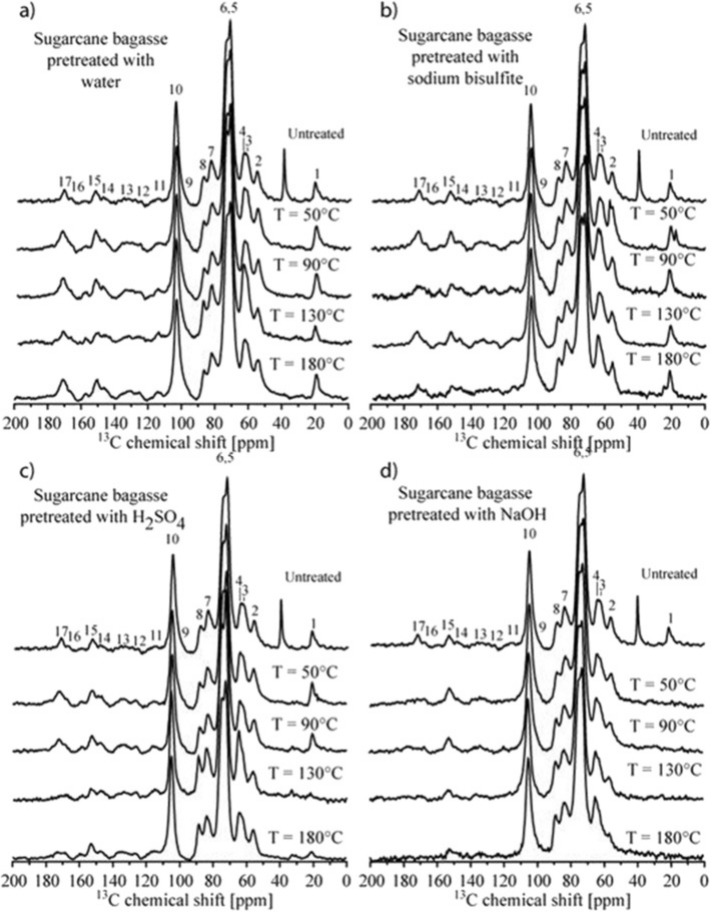
**CPMASTOSS spectra of the solid fractions of sugarcane bagasse sample submitted to the different pretreatments.(a)** hot water; **(b)** sodium bisulfite; **(c)** sulfuric acid and **(d)** sodium hydroxide pretreatments, respectively. Lines 3 and 7: C6 and C4 carbons from amorphous cellulose [38-42]; lines 4 and 8: C6 and C4 carbons [35-37]; lines 2, 11, 12, 13, 14: and 15: lignin carbons [37,43]; lines, 1, 3, 6, 7, 9 and 17: hemicelluloses carbons [36,44]; the unmarked line at 39 ppm is due to ash from biomass burned.

The effect of pretreatment on the feedstock compositions was also investigated using solid-state nuclear magnetic resonance (NMR). Figure 5 shows cross-polarization under magic angle spinning with total suppression of spinning sidebands (CPMASTOSS) spectra of the solid fractions of sugarcane bagasse samples submitted to the different pretreatments, which was very similar in all three novel grasses. All spectra were normalized with respect to line 10 (C1 carbon of cellulose). Chemical shift assignments based on the comparison with previously reported ^13^C NMR spectra of wood [35,36] and sugarcane bagasse [37] are listed in the caption of Figure 5 (see more complete attributions in table two of reference [37]).

The spectra of samples pretreated with hot water (Figure 5a) or sodium bisulfite (Figure 5b) at different temperatures were all similar to that of the untreated sample, showing that these pretreatments did not promote the efficient removal of hemicellulose and lignin, which is in agreement with the chemical composition analysis. Samples pretreated with sulfuric acid at temperatures up to 90°C also presented similar spectra to the untreated sample (Figure 5c). However, for pretreatment temperatures above 130°C there was a clear reduction in the hemicelluloses signals, lines 1 and 17, with little alteration in the lignin signals, lines 2, 11, 12, 13, 14 and 15. This suggests that, for sugarcane bagasse and the grasses, the pretreatment using sulfuric at 130°C acid is already effective for hemicellulose removal, but does not have a significant effect on lignin content. In samples pretreated with sodium hydroxide (Figure 5d), hemicellulose signals were already absent at 50°C whereas the lignin signals were reduced in line with an increase in pretreatment temperature. Indeed, the relative lignin content in the samples appeared similar for pretreatments at 130°C and 180°C, which suggests that the sodium hydroxide pretreatment of sugarcane bagasse and grasses at 130°C might be sufficient for the removal of hemicelluloses as well as effecting a reduction in lignin content.

NMR can also be used to give an indication of the composition of the crystalline cellulose to amorphous fraction after alkaline pretreatment. In feedstocks pretreated with sodium hydroxide at the higher pretreatment temperature (180°C), a decrease in the intensity ratio between lines 3 and 4 as well as between lines 7 and 8 was observed, which may be interpreted as a consequence of the removal of amorphous cellulose content by pretreatment.

**Figure 6:**
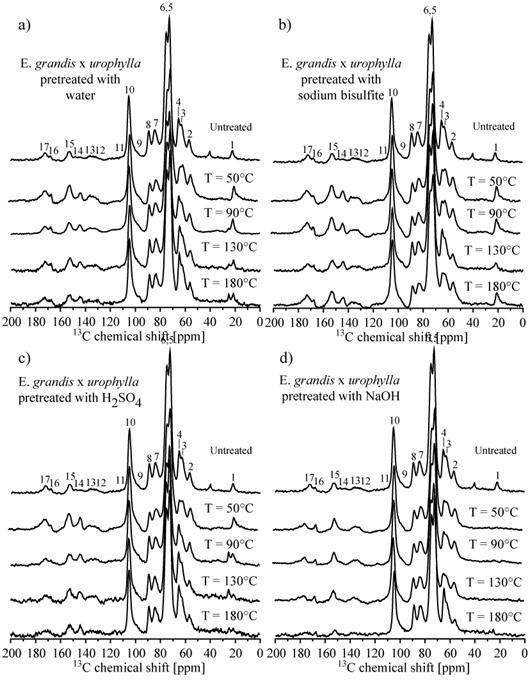
**CPMASTOSS spectra of the solid fractions of *E.grandis x urophylla *barks samples submitted to the different pretreatments.(a)** hot water; **(b)** sodium bisulfite; **(c)** sulfuric acid and **(d)** sodium hydroxide pretreatments, respectively.

NMR measurements were also carried out for the other grass feedstocks after distinct pretreatments and exhibited a response similar to that of sugarcane bagasse (data not shown). Conversely, NMR studies of the two types of eucalyptus bark show some particularities. Figure 6 shows CPMASTOSS spectra of the solid fractions of *E. grandis x urophylla* samples submitted to the different pretreatments.

As in the case of sugarcane bagasse and the other grasses samples, the spectra of *E. grandis x urophylla* samples pretreated with hot water (Figure 6a) or sodium bisulfite (Figure 6b) were very similar to that of the untreated sample, which confirms that these pretreatments are inefficient in the removal of hemicelluloses and lignin. Moreover, in samples pretreated with sulfuric acid (Figure 6c), the hemicellulose content was only decreased in response to pretreatment temperatures above 130°C, which was again very similar to the response of the novel grasses samples. However, sodium hydroxide was effective in the removal of hemicellulose at all pretreatment temperatures (Figure 6d), whereas lignin content was significantly reduced only in samples pretreated at 180°C. By contrast, pretreatment of sugarcane bagasse and the other investigated grasses at low temperatures appeared to be sufficient to reduce lignin content. Finally, the spectra of the sample pretreated with sodium hydroxide at 180°C also suggests that there was a reduction in the amorphous cellulose content in this sample after alkaline pretreatment. This should be compared to previously published results, using constant pretreatment temperature (120°), where higher sodium hydroxide concentrations (2% or 4%) and longer pretreatment times were required to remove the lignin fraction from these bark samples efficiently [15].

In summary, the NMR results indicated that, among the considered pretreatments, sulfuric acid was most effective in the removal of hemicellulose but sodium hydroxide was most efficient in the removal of hemicellulose together with a reduction in lignin content in both grasses and eucalyptus bark biomasses. However, the pretreatment temperature was also an important parameter and the use of higher temperatures promoted the removal of amorphous cellulose. In this sense, the results point to the intrinsic advantages of grass samples, which require lower pretreatment temperatures than eucalyptus barks.

### Soluble fraction analysis: monosaccharide and furaldehyde content

**Figure 7:**
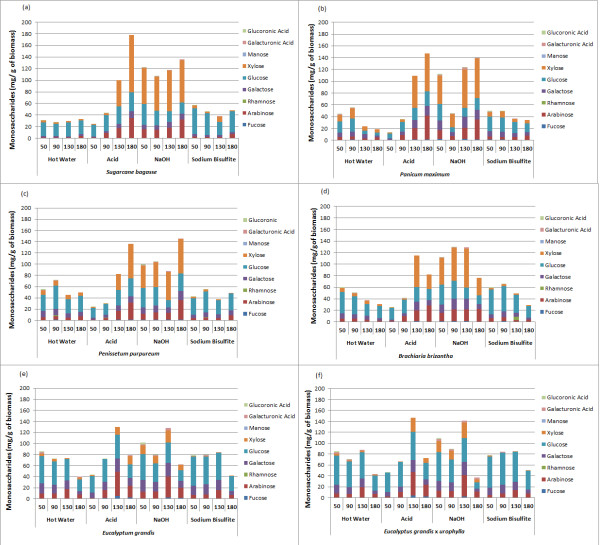
**Monosaccharide composition in the liquor fraction from different pretreatments. (a)** Sugarcane bagasse; **(b)***Panicum maximum*; **(c)***Pennisetum purpureum*; **(d)***Brachiaria brizantha*; **(e)***Eucalyptus grandis* bark; **(f)***E. grandis x urophylla* bark. Pretreatment types and temperatures are indicated.

**Figure 8:**
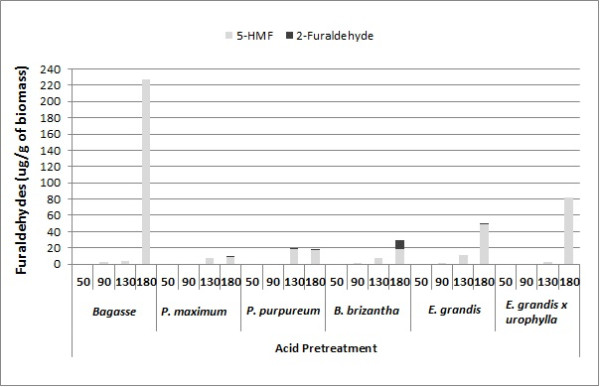
2-furaldehyde and 5-hydroxymethyl-furfural content in the liquor fraction from acid pretreatment at increasing temperatures, ranging from 50°C to 180°C.

To evaluate the generation of inhibitors and potential valuable products in the soluble phase of the protocol, a profile of compounds moved by the pretreatment solution was determined. The monosaccharide composition of the soluble fraction from hot water, sulfuric acid, sodium hydroxide and sodium bisulfite pretreatments at increasing temperatures, ranging from 50°C to 180°C (Figure 7), was studied. The potential formation of 2-furaldehyde and 5-hydroxymethyl-furaldehyde as a result of sulfuric acid pretreatment was also investigated in all six feedstocks (Figure 8).

For the pretreatments conducted at 50°C, glucose was the main monosaccharide in the soluble fraction from most of the biomasses and was detected together with xylose and other hemicellulose sugars (Figure 7). It can be related to an easier solubilization of glucose from hemicellulose, as well as the removal of the amorphous cellulose fraction. This enrichment in glucose was particularly evident in hot water, acid and sulfite pretreatments. In the soluble fraction from sodium hydroxide pretreatment, the xylose amount was higher than glucose for all grasses even at 50°C, while for the bark samples the opposite was observed. This difference is associated with the efficient removal of the hemicellulose fraction by alkaline pretreatment, even at lower temperature, and the different composition of hemicelluloses in eucalyptus bark, which has a lower content of xylans.

With increasing temperatures, a gradual increase of xylose, arabinose, galactose and other monosaccharides was also observed for all pretreatments, indicating an efficient removal of the hemicellulose fraction. However, acid and alkaline pretreatments indicated a higher content of monosaccharides in the soluble fraction for all biomasses.

At higher temperature (180°C), a decrease of glucose content for all biomasses, in spite of xylose increase, became evident, most notably with acid pretreatment. The fall in glucose observed at higher temperatures can be explained by the formation of inhibitors, as shown in Figure 8. The highest 5- hydroxymethyl-furfural content was found for all biomasses pretreated at 180°C using sulfuric acid. However, lower amounts could be observed at 90°C or higher. Acidic conditions lead to a rapid decay of glucose into 5-hydroxymethyl-furfural by dehydration [45]. Sugarcane bagasse and bark were more susceptible to cellulose dehydration, whereas the perennial grasses showed levels below 20 μg of hydroxymethyl-furfural per gram of biomass. C5 conversion into 2-furaldehyde was found mainly in the soluble fraction from perennial grasses, with *B. brizantha* being most prone to the production of 2-furaldehyde under acid treatment.

### Morphological changes produced by pretreatments

To evaluate the effect of pretreatments on the morphology of different biomasses to improve enzymatic digestibility, we used scanning electron microscopy to investigate the morphological changes produced by sodium hydroxide pretreatment at 130°C. This pretreatment results in significant lignin and hemicellulose removal and, consequently, a higher cellulose enrichment, without the production of high levels of inhibitor.

**Figure 9:**
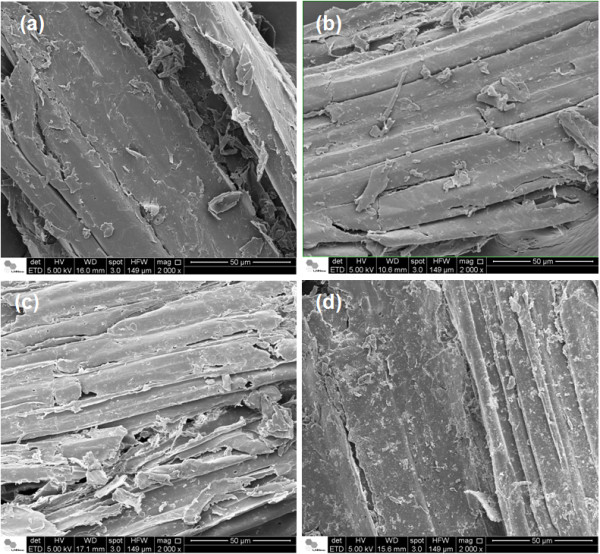
**Scanning electron microscopy images of sugarcane bagasse before and after different pretreatments at 130°C. (a)** Raw sugarcane bagasse (no pretreatment); **(b)** sugarcane bagasse pretreated with hot water; **(c)** sugarcane bagasse after sulfuric acid pretreatment and **(d)** sugarcane bagasse obtained after sodium bisulfite pretreatment.

Figure 9 shows the effects of different pretreatments on sugarcane bagasse, compared to raw material. A sample obtained from hot water pretreatment (Figure 9b) showed a similar surface to that obtained for raw bagasse (Figure 9a), where there was a continuous covering layer (possibly formed by lignin and hemicellulose). After acid pretreatment (Figure 9c), cellulose bundles were more evident, with less cohesion between them. This can be associated with the high level of hemicellulose removal, thereby enabling enzyme access to the cellulose fiber. A continuous layer over the cellulose bundles surface was also observed after sodium bisulfite treatment, but in this case some parts of the bundles were already evident, as shown in Figure 9d. Furthermore, it was possible to observe some residues over the surface, which could be associated with lignin modification and precipitation.

**Figure 10:**
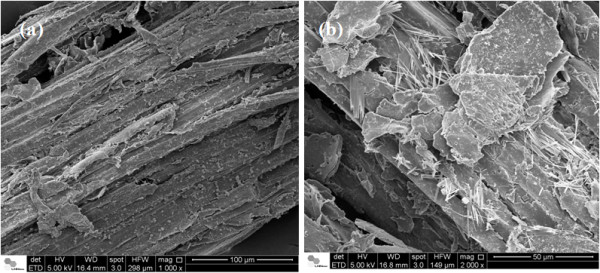
**Scanning electron microscopy images of sugarcane bagasse submitted to sodium hydroxide pretreatment. (a)** General view of sugarcane surface after alkali pretreatment and **(b)** higher magnification of cellulose whiskers on biomass surface.

Among the pretreatments described here, the largest morphological changes were produced by sodium hydroxide. Figure 10 shows the effects of sodium hydroxide on sugarcane bagasse at 130°C, demonstrating the removal of the covering layer, mainly lignin (as determined by chemical composition), and a consequent loss of biomass structure, with separation of fiber bundles (Figure 10a). Lignin precipitation was also observed on the surface of fibers. At higher magnification, the presence of microfibrillated cellulose on the surface of samples could be observed (Figure 10b). Recently, such cellulose particles have been the focus of an exponentially increasing number of works, mainly interested in their structure and their potential to act as fillers to improve mechanical and barrier properties of biocomposites. Cellulose nanofillers are mainly native cellulose (cellulose I), extracted by traditional bleaching treatments of lignocellulosic fibers. However, the extraction conditions (time, temperature, chemical concentration) are fundamental to the efficient extraction of cellulose nanoparticles with the required characteristics [46]. These microfibrillated celluloses were not observed for sugarcane bagasse in a previous pretreatment condition using the same 1% sodium hydroxide concentration when preceded by a sulfuric acid pretreatment step at 120°C and a residence time for the alkaline step of 1 h [37]. In the present paper, however, the residence time was 40 min and treatment temperature was 130°C, without the acid step.

**Figure 11:**
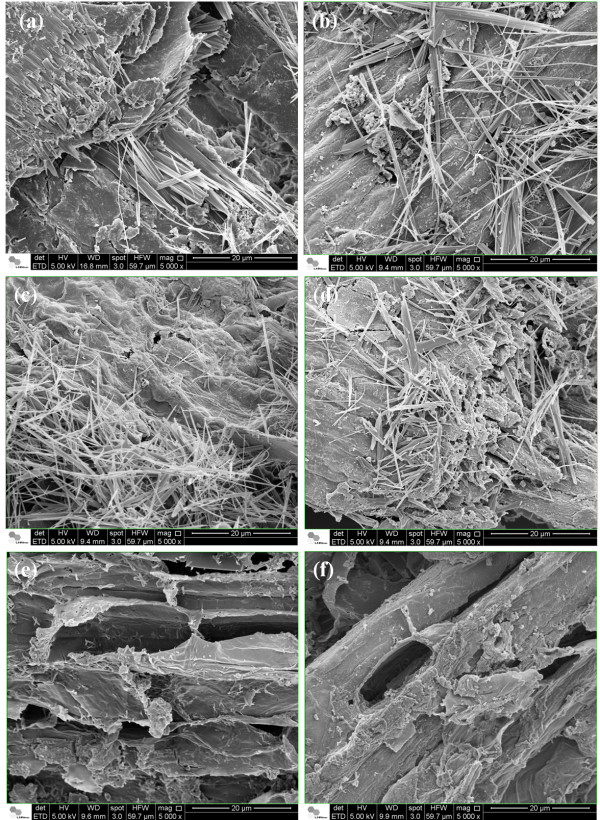
**Scanning electron microscopy images of different biomasses pretreated with sodium hydroxide at 130°C, revealing the production on cellulose whiskers. (a)** sugarcane bagasse; **(b)***Panicum maximum*; **(c)***Pennisetum purpureum*; **(d)***Brachiaria brizantha*; **(e)***Eucalyptus grandis* bark; and **(f)***E. grandis x urophylla* bark.

The effects of sodium hydroxide pretreatment at 130°C were also studied in the other biomass samples (Figure 11). Scanning electron microscopy images of the perennial grasses also revealed longer and isolated fibers of crystalline cellulose, compared to those of sugarcane bagasse (Figure 11a-d). This indicates their potential for the generation of natural fillers after efficient enzymatic hydrolysis, when the crystalline cellulose can be easily accessed and cleaved by cellulases. The surface images also revealed a notable effect of sodium hydroxide pretreatment at 130°C on eucalyptus bark surface, mainly due to the removal of lignin and its precipitation. However, microfibrillated cellulose was not detected on the barks surface, suggesting the need for more severe pretreatment conditions to obtain pure cellulose fibers.

### Changes in enzymatic saccharification

**Figure 12:**
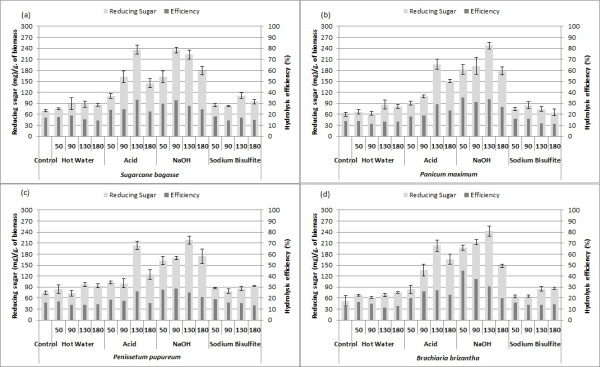
**Automated enzymatic saccharification of raw biomasses and pretreated samples. (a)** Sugarcane bagasse; **(b)***Panicum maximum*; **(c)***Pennisetum purpureum*; **(d)***Brachiaria brizantha*; **(e)***Eucalyptus grandis* bark; **(f)***E. grandis x urophylla* bark. Pretreatment types and temperatures are shown.

Saccharification screening was performed to verify the effect of pretreatments on the saccharification potential of different biomasses. Results of this analysis indicated that sulfuric acid and sodium hydroxide greatly improved the sugar release from sugarcane bagasse and the three perennial grasses, whilst for the eucalyptus bark samples, sodium hydroxide pretreatment was significantly better (Figure 12). This differential effect could be related to the different hemicelluloses and different composition of lignin in eucalyptus bark.

The amounts of sugar released by the six feedstocks pretreated with hot water and sodium bisulfite were very similar to that of the control, and for all biomasses there was only a discrete effect of increased temperature, which correlates well with the results of the chemical analysis. However, the increase of pretreatment temperature significantly affected the enzymatic digestibility of sugarcane bagasse and grasses submitted to acid and sodium hydroxide. A gradual increase of sugar release was observed up to 130°C, followed by a decrease at 180°C. For the eucalyptus barks, the temperature effect was also very discrete, and even at the lower temperatures used, the glucose amount released was relatively high in both feedstocks.

## Conclusion

The biomass feedstocks investigated in this work illustrate potential as a source of carbohydrates for bioethanol production. These feedstocks can be sustainably grown and applied to local production during the inter-season, when no sugarcane bagasse is produced. Alkaline pretreatment at 130°C led to higher saccharification yields for the grass feedstocks, showing quite similar amounts of reducing sugars released for the three grasses and sugarcane bagasse. The alkaline treatment also resulted in higher glucose release for eucalyptus bark, even at the lower temperatures used. The relatively higher sugar yields obtained from sugarcane bagasse and the grasses, when compared to eucalyptus bark, can be explained by the morphological and chemical changes occurring during pretreatment. Chromatographic analysis indicated a higher cellulose enrichment in the grasses after sodium hydroxide pretreatment, and morphological analyses by scanning electron microscopy illustrated the effects of pretreatment on biomass structure, specifically the removal of lignin and the production of microfibrillated cellulose in grass samples, which justifies the documented improvement in enzymatic digestibility.

## Material and methods

### Plant material

Sugarcane bagasse obtained after the industrial process for juice extraction for ethanol generation was kindly provided by the Cosan Group (Ibaté, SP, Brazil). Bark residues from mechanized harvesting of two commercial Eucalyptus clones (*E. grandis* and the hybrid *E. grandis x urophylla*) were provided by the Suzano Pulp and Paper Company (Itapetininga, SP, Brazil). Grass biomasses (*Pennisetum purpureum*, *B. brizantha* and *Panicum maximum*) grown for 180 days were supplied by Embrapa (Empresa Brasileira de Pesquisa Agropecuária). All the biomasses were dried in a convection oven at 60°C for 24 h and then ground to a fine powder in a ball mill (TissueLyser II, Qiagen (Hilden, Germany) for 2 min at 25 Hz.

### Determination of silicon level by x-ray fluorescence spectrometry

XRF measurements were performed as previously described [47], using a commercial Portable XRF instrument (Niton XL3t900 Analyzer, Thermo Scientific, Hemel Hempstead,

Hertfordshire, UK) equipped with an X-ray tube and a silicon drift detector. All measurements were carried out in a helium atmosphere with a helium flow rate of 70 centiliters min^-1^, and the samples were exposed to X-rays for 30 s (instrument settings: main range: 5 s, 6.2 kV, 100 uA, filter blank (no physical filter in the primary beam); low range: 25 s, 6.2 kV, 100 uA, filter blank). XRF experiments were performed on the raw powder biomasses, previously dried at 60°C for 24 h, and silica powder (Fisher Scientific, Loughborough, UK; product number S/0680/53) was used as standard to the calibration curve generation. To obtain a repeatable photon flux from the sample to the XRF detector, sample pellets were prepared using approximately 0.7 g of ground plant material, submitted to 11 tons for 2 s using a manual hydraulic press (Specac, Orpington, UK). Cylindrical pellets of around 5 mm thickness and 12 mm diameter were obtained and measured on both surface sides.

### Immunolabeling of hemicellulosic polysaccharides

The hemicelluloses fraction from biomasses (30 mg, excluding the soluble fraction) was obtained by extraction with 4 mL of 4 M sodium hydroxide/1% sodium borohydride for 1 h at room temperature. The extraction procedure was repeated twice and the supernatants were combined and neutralized with acetic acid. The polysaccharides from the hemicellulose fraction were precipitated with ethanol and solubilized in water (10 mL). Following protocol optimization, specific polysaccharide dilutions were used for each antibody: LM10 (40× dilution for all biomasses); LM11 (40× dilution for all biomasses); and LM21 (20× dilution for all biomasses). Monoclonal antibodies were obtained as hybridoma cell culture supernatants from Plant Probe Laboratories (Leeds, UK) and diluted 25-fold for all assays. Selected antibodies were: LM10 and LM11, which recognize a xylan and arabinoxylan epitope respectively; and LM21, which primarily recognizes mannan, galactomannan and glucomannan polysaccharides [30-32]. Stock solutions (10 μg/mL in deionized water) of beech wood xylan (x4252; Sigma-Aldrich, Seelze, Germany), wheat arabinoxylan (70502a; Megazyme, Bray, County Wicklow, Ireland) and Konjac glucomannan (50601; Megazyme) were used as positive control for LM10, LM11 and LM21, respectively. The immunolabeling assays were performed as previously described and the degree of antibody binding was measured at 620 nm after a reaction time of 40 min to enable color development.

### Pretreatment

Plant materials (400 mg) were pretreated in small-scale bombs, using 16 mL of pretreatment solution (hot water, 0.1 M sulfuric acid, 0.25 M sodium hydroxide and 3% (w/v) sodium bisulfite). Pretreatment was performed for 40 min in the oven at different temperatures (50°C, 90°C, 130°C and 180°C). After cooling to the room temperature, samples were transferred to 50 mL centrifuge tubes and centrifuged at 4,000 g for 15 min. The liquor fraction was recovered into a new tube and stored for further analysis. The solid fraction was resuspended in 5 mL of ethanol, then homogenized and centrifuged at 4,000 g for 15 min. An ethanol washing procedure was conducted in triplicate before the biomass was dried at room temperature.

### Chemical analysis

#### Soluble extraction

The soluble content was determined by sequential extraction with different organic solvents. Powdered plant material (200 mg) was weighed into a centrifuge tube and extracted with phenol (5 mL), under constant vortex agitation for 1 min. Samples were centrifuged for 20 min at 10,000 g, after which the supernatant was discarded. The pellet was resuspended in 10 mL of a chloroform:methanol (2:1 v/v) solution and homogenized vigorously for 2 min. The sample was centrifuged as previously and the pellet was washed with ethanol (2 mL) twice more. After washing, 5 mL of 90% dimethyl sulfoxide was added to each sample, which was then and left rocking overnight at room temperature. The dimethyl sulfoxide supernatant obtained by centrifugation was removed and the solid fraction was washed three times with ethanol absolute. Finally, samples were dried in a vacuum dryer and their dry weight was recorded. The difference between the initial and final weights was used to determine the soluble fraction (%).

#### Digestion of non-crystalline polysaccharides for monosaccharide analysis

Pretreated samples and soluble extracted biomass samples (10 mg) were weighed in a 2 mL capped tube and incubated in 0.5 mL of 2 M trifluoroacetic acid (TFA) for 4 h at 100°C in an argon atmosphere. Samples were homogenized every hour, then the TFA was evaporated in a centrifugal evaporator at 45°C. Samples were resuspended in 0.5 mL of Miliq water under vigorously agitation and centrifuged at 10,000 g for 10 min. The supernatant was recovered into a new tube without disturbing the pellet and the washing procedure repeated once. The water soluble monosaccharides in the supernatant (hemicellulose fraction) were vacuum-dried and stored at room temperature for further chromatographic analysis. The remaining solid fraction was used to determine the crystalline cellulose content.

#### Crystalline cellulose content

The residual solid fraction obtained after TFA extraction (as described above) was initially submitted to hydrolysis using 1 mL of Updegraff reagent [48]. Samples were vortexed then incubated at 100°C for 30 min. After cooling to room temperature, samples were centrifuged and the supernatant was carefully discarded without disturbing the remaining crystalline cellulose fraction. The pellet was washed with 1.5 mL of water and centrifuged. Three additional washes were performed using 1.5 mL of acetone before the samples were dried at room temperature. The hydrolysis of crystalline cellulose was performed at room temperature for 30 min, using 175 μL of 72% (v/v) sulfuric acid. The reaction was stopped by the addition of 825 μL of water and then the sample was vortexed. Finally the sample was centrifuged before the glucose content was determined by the anthrone method [49].

#### Acetyl bromide soluble lignin

The total lignin content of soluble extracted plant material and samples obtained from each pretreatment condition was determined using the acetyl bromide method [50]. Powder biomass (3.5 mg) was weighed into a 2 mL cap tube and 250 μL of freshly prepared acetyl bromide solution (25% v/v acetyl bromide/glacial acetic acid) was added. Samples were incubated at 50°C for 3 h, with periodical agitation. After cooling to room temperature, the hydrolysate was transferred to a 5 mL volumetric flask. One mL of 2 M sodium hydroxide was added to the 2 mL tube to generate the acetyl bromide excess before transfer to a volumetric flask. Next, 175 μL of hydroxylamine-hydrochloric acid was added to each sample, which were then vortexed vigorously. Finally, the volume was adjusted to 5 mL with glacial acetic acid and the absorbance was measured at 280 nm. The acetyl bromide soluble lignin (%) was determined using the extinction coefficient for grasses ( 17.75 ).

#### Monosaccharide analysis

Monosaccharide analysis was performed by high performance anion-exchange chromatography (Dionex IC 2500; Thermo Scientific, Camberley, Surrey, UK) on a Dionex Carbopac PA-10 column with integrated amperometry detection [51]. The separated monosaccharides were quantified using external calibration with an equimolar mixture of nine monosaccharide standards (arabinose, fucose, galactose, galacturonic acid, glucose, glucuronic acid, mannose, rhamnose and xylose), which were subjected to TFA hydrolysis in parallel with the samples.

### Sugar content in pretreatment liquors

Monosaccharide composition in the liquor fraction obtained from each pretreatment condition was also determined by ion chromatography. Acidic liquor samples were initially neutralized with barium hydroxide solution (150 mM), followed by barium carbonate powder. Alkaline samples were neutralized with 2 M hydrochloric acid. All the samples were adjusted to the same final volume and centrifuged at 4,000 g to warranting precipitate removal. Sulfite liquor samples were filtered using an ion exchange column (onGuard II Ba Cartridge, Dionex) to eliminate the residual ions from the samples.

The neutralized liquor fraction (1 mL) was transferred to a microcentrifuge tube and vacuum. Hydrolysis using 500 μL of 2 M TFA was carried out at 100°C for 4 h. After cooling to room temperature, samples were dried and twice washed with 200 μL of isopropanol. Monosaccharide content was determined as previously described.

### Analysis of furfural and 5-hydroxymethyl-furfural content

Liquor fractions obtained from each pretreatment condition were neutralized and subjected to chromatography using a Luna® 5 μm C18(2) 100 Å LC Column 150 × 4.6 mm, together with a C18 4 × 2.0 mm ID guard column (both from Phenomenex, Cheshire, UK) to verify furfural and 5-hydroxymethyl-furfural content. Analyses were carried out using a Surveyor HPLC (Thermo Scientific, Hemel Hempstead, UK), with an elution system of acetonitrile by reversed-phase in an isocratic gradient (5% acetonitrile and 95% deionized water) at 1 mL/min. The eluted furfuraldehydes were detected by UV absorbance at 284 nm using a Finnigan Surveyor PDA Plus detector (Thermo Scientific; Hemel Hempstead, UK). The furfurals were quantified by interpolation of a calibration curve within the range of 0.005 μg/mL to 50 μg/mL in water.

### Solid-state nuclear magnetic resonance

NMR experiments were performed in a Varian INOVA spectrometer (Varian; Palo Alto, California, USA) operating at ^13^C and ^1^H frequencies of 100.5 and 400.0 MHz, respectively. A Varian 5-mm magic angle spinning double-resonance probe head was used. The spinning frequency of 4.5 kHz was controlled by a Varian pneumatic system ensuring a rotation stability of approximately 2 Hz. CPMASTOSS was used for ^13^C excitation in all experiments. Typical π/2 pulse lengths of 3.5 μs (^13^C) and 4.5 μs (^1^H), continuous wave proton decoupling with field strength of 60 kHz, cross-polarization time of 1 ms and recycle delays of 2 s were used .

### Scanning electron microscopy

Surface images of the grasses and both eucalyptus barks after variable pretreatment conditions were obtained by scanning electron microscopy and compared with the raw material. Milled samples were critical point dried before coating with gold in a Balzers SCD 050 sputter coater (BAL-TEC AG, Balzers, Liechtenstein). Samples were viewed using a scanning electron microscope model Quanta 650-FEG (FEI, Hillsboro, Oregon, USA) from the National Laboratory of Nanotechnology (LNNano-CNPEM) in Campinas, SP, Brazil. A large number of images were obtained from different areas of the samples (at least 20 images per sample) to confirm the reproducibility of results.

### Automated enzymatic saccharification analysis

Automated saccharification assays were performed as previously described [52]. Powder pretreated samples and soluble extracted biomasses were dispensed into 96-well plates using a custom-made robotic platform (Labman Automation Ltd., Stokesley, UK) and the standard target weight of material was 4 mg. Enzymatic hydrolysis and sugar determination were performed automatically using a robotic platform (Tecan Evo 200; Tecan Group Ltd., Männedorf, Switzerland). The hydrolysis was carried out in a monitored shaking incubator (Tecan Group Ltd.) using an enzyme cocktail with a 4:1 ratio of Celluclast and Novozyme 188 (cellobiase from *Aspergillus niger*; both from Novozymes, Bagsvaerd, Denmark) at 30°C for 8 h. The saccharification of the powdered biomass was performed in a total volume of 600 μl for 8 h, with an enzyme loading of 8 FPU/g of biomass. Automated determination of released reducing sugar after hydrolysis was performed using 3-methyl-2-benzothiazolinone hydrozone, as previously described and established [52,53].

## Abbreviations

CPMASTOSS: cross-polarization under magic angle spinning with total suppression of spinning sidebands; ELISA: enzyme-linked immunosorbent assay; NMR: nuclear magnetic resonance; TFA: trifluoroacetic acid; XRF: X-ray fluorescence spectroscopy.

## Competing interests

The authors declare that they have no competing interests.

## Authors’ contributions

MAL planned the conceptual process, performed all the experiments and was responsible for results analysis and manuscript draft. LDG helped with the conceptual process, monosaccharide analysis, results discussion and manuscript draft. CGSK helped with immunolabeling assays and analysis and manuscript draft. RS was responsible for the robotic platforms operation during the saccharification assays and helped on the results analysis. CAR conducted the scanning electron microscopy experiments and analysis. CAL contributed to the eucalyptus barks preparation, analysis and evaluation of results. MAC prepared grass samples and made them available for this study. ERA and ODB performed the NMR experiments and analysis, and contributed to manuscript draft. IP and SMM coordinated the overall study, and contributed to results analysis and writing up the paper. All the authors approved the final manuscript.

## Supplementary Material

Additional file 1 **Surface images obtained by scanning electron microscopy showing the silica bodies (phytoliths) on plant tissue.** (a) *Panicum maximum*; (b) *Pennisetum purpureum* and (c) *Brachiaria brizantha.* Click here for file
