# Supplementary material for: Evaluating the composition and processing potential of novel sources of Brazilian biomass for sustainable biorenewables production
Source: Biotechnol Biofuels. 2014 Jan 18;7:10. doi: 10.1186/1754-6834-7-10 (PMC4028816; doi:10.1186/1754-6834-7-10)

SUPPLEMENTARY FILE 1

Surface images obtained by SEM showing the silica bodies (phytoliths) on plant tissue. (a) *P. maximum*; (b) *P. purpureum* and (c) *B. brizantha*.

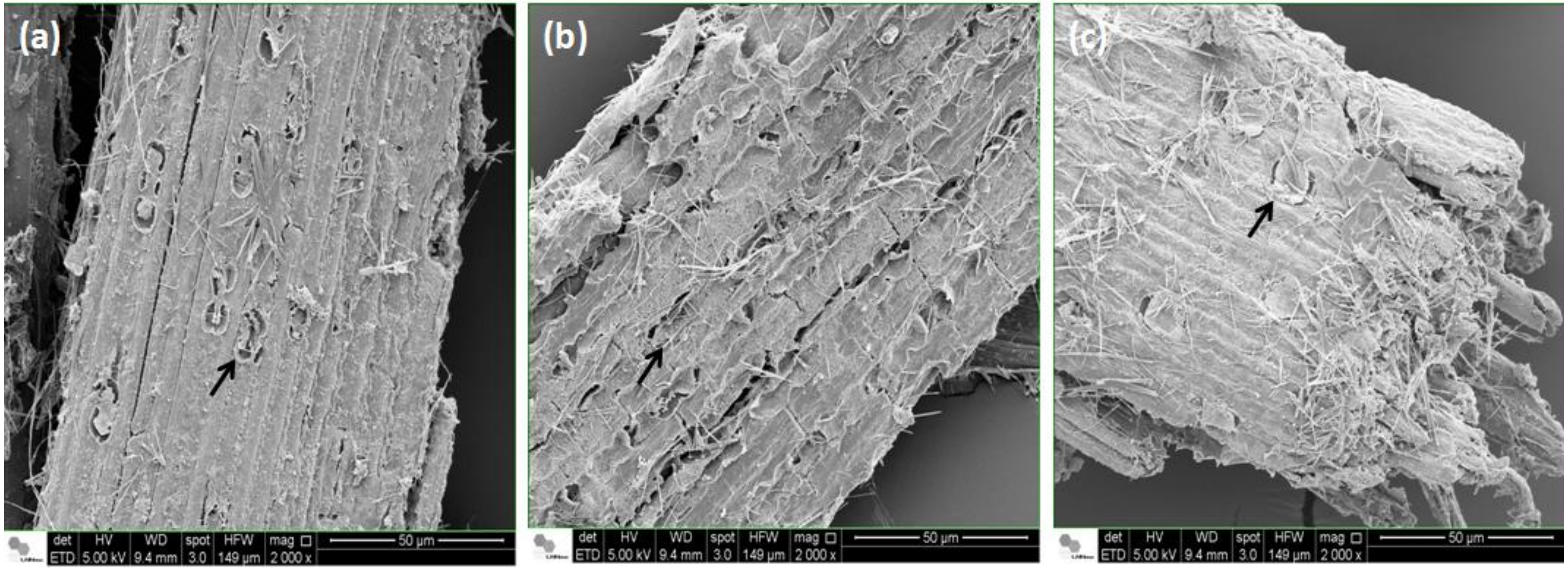

Supplement: Additional file 1 — Surface images obtained by scanning electron microscopy showing the silica bodies (phytoliths) on plant tissue. (a) Panicum maximum; (b) Pennisetum purpureum and (c) Brachiaria brizantha. [file 1754-6834-7-10-S1.pdf]
